# Mechanism of *Radix Bupleuri* and *Hedysarum Multijugum Maxim* drug pairs on liver fibrosis based on network pharmacology, bioinformatics and molecular dynamics simulation

**DOI:** 10.1371/journal.pone.0318336

**Published:** 2025-01-27

**Authors:** Lefei Yu, Pan Yu, Yongchang Cao, Weiya Cao

**Affiliations:** 1 College of Computer Science and Cyber Security (Oxford Brookes College), Chengdu University of Technology, Chengdu, China; 2 School of Public Health, Anhui University of Science and Technology, Hefei, China; Guangdong Nephrotic Drug Engineering Technology Research Center, Institute of Consun Co. for Chinese Medicine in Kidney Diseases, CHINA

## Abstract

A number of studies demonstrate the therapeutic effectiveness of *Radix Bupleuri* (RB) and *Hedysarum Multijugum Maxim* (HMM) in treating liver fibrosis, but the exact molecular mechanisms remain unclear. This study aims to explore the mechanism of RB-HMM drug pairs in treating liver fibrosis by using network pharmacology, bioinformatics, molecular docking, molecular dynamics simulation technology and in vitro experiments. Totally, 155 intersection targets between RB-HMM and liver fibrosis were identified. In the protein-protein interaction (PPI) network, the top 10 hub targets with the highest node connection values were TNF, IL-6, AKT1, EGFR, HIF1A, PPARG, CASP3, SRC, MMP9 and HSP90AA1. GO functional and KEGG pathway enrichment analysis involved 335 biological processes, 39 cellular components, 78 molecular functions, and 139 signaling pathways. The bioinformatics analysis indicated that TNF, IL-6, PPARG and MMP9 were promising candidate genes that can serve as diagnostic and prognostic biomarkers for liver fibrosis. Moreover, the molecular docking and molecular dynamic simulation of 50 ns well complemented the binding affinity and strong stability between the three common compounds MOL000098 (quercetin), MOL000354 (isorhamnetin) and MOL000422 (kaempferol) and four final hub targets (TNF, IL-6, PPARG and MMP9). Calculation of binding free energy and decomposition free energy using MM_PBSA and MM_GBSA also validated the strong binding affinity and stability of 12 systems. MOL000098 (quercetin) was selected via MTT assay and western blot assay verified MOL000098 (quercetin) treatments remarkably decreased the protein levels of TNF and IL-6 in TGFβ stimulated LX2 cells. In conclusion, RB-HMM drug pairs can affect the progression of liver fibrosis through multiple components, multiple targets and multiple pathways, and treat liver fibrosis possibly through anti-inflammatory and affecting cell apoptosis.

## Introduction

Excessive accumulation of extracellular matrix components, known as liver fibrosis, is a serious medical issue that can lead to liver cirrhosis and hepatocellular carcinoma, highlighting the crucial need for effective therapeutic interventions [1]. In this therapeutic context, natural products, particularly flavonoids, have attracted significant attention due to their potential to alleviate liver fibrosis [2]. The combined use of herbal components, known as "drug pairs" in the Traditional Chinese Medicine (TCM) framework, has shown significant therapeutic effectiveness [3]. This review offers a detailed analysis of the molecular mechanisms behind the anti-fibrotic effects of TCM and highlights potential therapeutic targets for liver fibrosis [4].

However, the intricate mechanisms underpinning the therapeutic effects of these drug pairs, particularly in the context of liver fibrosis, remain largely enigmatic [5]. Empirical and clinical evidence has corroborated the potent anti-fibrotic attributes of *Radix Bupleuri* (RB) and *Hedysarum Multijugum Maxim* (HMM), independently. RB had the hepatoprotection function on acetaminophen-induced liver injury and the mechanisms of Chaihu-Shugan-San against liver fibrosis were explored by integrated multi-omics and network pharmacology approaches, highlighting its therapeutic potential [6, 7]. The main active ingredients of HMM including astragaloside, astragalus flavone and astragalus polysaccharide and the anti-liver fibrosis effects and possible mechanisms were evaluated in animal models through systematic review and meta-analysis [8]. In addition, the action mechanism of immune cells was also explored by which HMM exerted its anti-fibrotic effects, providing insights into the bioactive components and potential targets [9]. The synergistic potential of their combined application, alongside the detailed mechanisms facilitating their cooperative action in the suppression of liver fibrosis, warrants comprehensive elucidation.

To identify bioactive ingredients and potential targets of RB-HMM, the integration of advanced methodologies such as network pharmacology, bioinformatics, molecular docking, and molecular dynamics simulation has become indispensable. Network pharmacology can be applied to analyze the potential mechanism of TCM in treating liver fibrosis, revealing the multi-target and multi-pathway nature of its therapeutic effects [10]. This bioinformatics study explored the anti-fibrotic effects of the only currently-approved drugs Pirfenidone and Nintedanib, identifying novel TGFβ1 regulated key genes and pathways that may be targeted by these drugs [11]. This study used molecular docking and network pharmacology to investigate the pharmacological basis of *Isodon ternifolius* against liver fibrosis, contributing to the understanding of the therapeutic mechanism [12]. In addition, this article used molecular dynamics simulation combining machine learning and experimental validation to investigate the interaction between acetylbinankadsurin A (ACBA) and proteins associated with liver fibrosis, providing insights into the stability of these complexes and their potential therapeutic significance [13]. These cutting-edge techniques facilitate a holistic exploration of drug-target interactions and enable predictions regarding potential therapeutic efficacies.

This study endeavors to systematically identify the potential target proteins associated with liver fibrosis that are modulated by the RB-HMM drug pairs through extensive database analysis. Utilizing Gene Ontology (GO) and Kyoto Encyclopedia of Genes and Genomes (KEGG) pathway enrichment analyses, we will construct a comprehensive network that delineates the relationships between the drug, its bioactive constituents, the targeted proteins, and the disease pathology. By incorporating advanced bioinformatic analytical strategies including microarray analysis, survival analysis, and tumor staging, we aim to pinpoint the core targets that are pivotal to the anti-fibrotic activities of this drug pair. Molecular docking and molecular dynamics simulations will be deployed to rigorously assess the binding affinities of the bioactive constituents to the identified core targets and to evaluate the stability of the resultant complexes.

**Fig 1:**
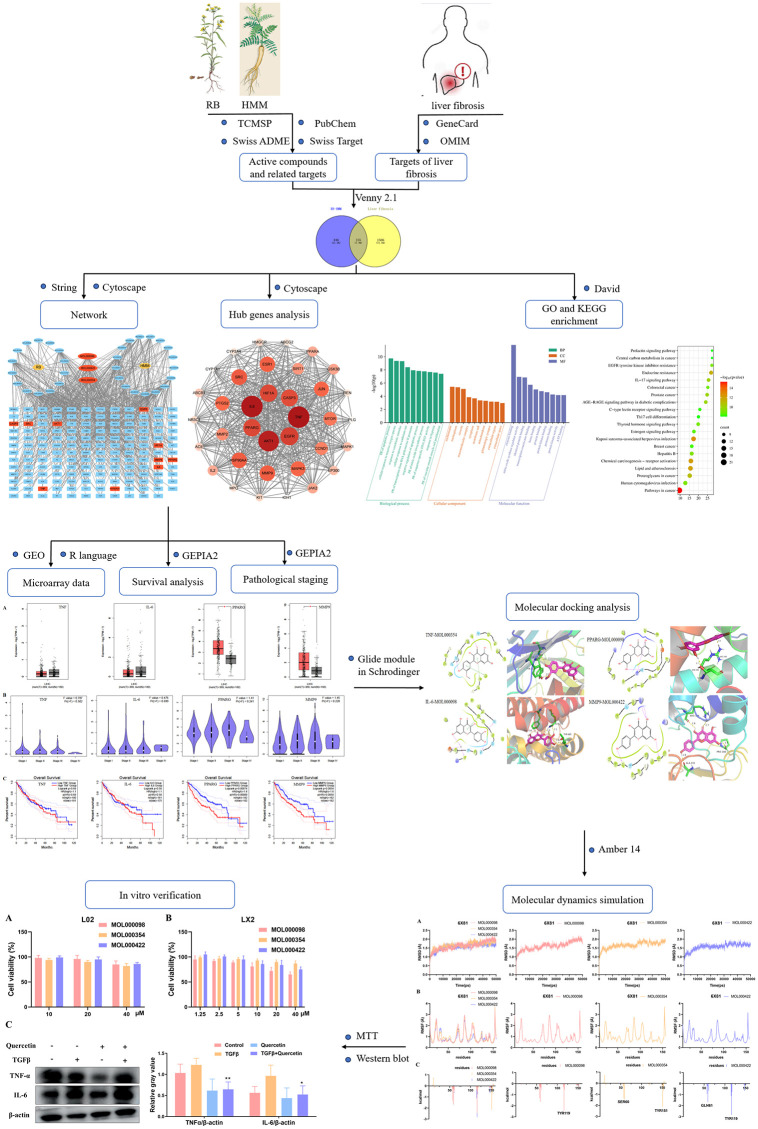
The flow chart.

In conclusion, this investigation will harness the synergistic power of network pharmacology, bioinformatics, molecular docking, and molecular dynamics simulation to systematically unravel the therapeutic mechanisms of the RB-HMM combination in the context of liver fibrosis. The flow chart of the entire study is shown in Fig 1. This endeavor will not only elucidate potential bioactive ingredients and therapeutic targets but will also contribute significantly to the theoretical foundation for the application of TCM principles in the clinical management of liver fibrosis, thereby advancing the field of hepatology and therapeutics.

## Materials and methods

### Active compounds and related targets collection of *Radix Bupleuri* (RB) and *Hedysarum Multijugum Maxim* (HMM)

The potential active compounds of RB and HMM were identified using the Traditional Chinese Medicine Systems Pharmacology Database and Analysis Platform (TCMSP, http://lsp.nwsuaf.edu.cn/) and Swiss ADME platform (http://www.swissadme.ch/) [14, 15]. Active compounds were screened based on oral bioavailability (OB) ≥30% and drug-likeness (DL) ≥0.18 [16]. The SMILES structures of the selected active ingredients were retrieved from the PubChem database (https://pubchem.ncbi.nlm.nih.gov/) to create a molecular library [17]. Potential targets were predicted using the 2D/3D structural similarity principle and the Swiss Target Prediction platform (http://www.swisstargetprediction.ch/), with duplicate targets removed [18].

### Gene targets of liver fibrosis collection

A keyword like “liver fibrosis” was entered into the GeneCard database (https://www.genecards.org/) and OMIM database (https://omim.org/) [19, 20]. Disease-related target information was extracted and subsequently imported into the UniProt database (https://www.uniprot.org/) to retrieve the corresponding gene names and gene IDs [21]. Furthermore, the overlapping targets associated with compounds (RB, HMM) and liver fibrosis were identified using Venny 2.1 (https://bioinfogp.cnb.csic.es/tools/venny) [22].

### Protein-protein interaction (PPI) study

The overlapped targets were then imported into the STRING database (https://cn.string-db.org/) database to construct the PPI network [23]. The resulting PPI data were further analyzed in Cytoscape 3.9.1 to assess the topological properties of the intersecting targets, including the calculation of node connectivity and centrality measures [24]. Hub target genes were identified using three algorithms: Degree, Closeness, and Betweenness. For this analysis, Homo sapiens was selected as the species, and all other parameters were kept at their default settings.

### GO and KEGG analysis

The GO function and KEGG pathway were performed using the online David (https://david.ncifcrf.gov/) database, with a significance threshold of P < 0.05 [25]. The top 10 biological functions including cellular components (CC), molecular functions (MF), and biological processes (BP), as well as the top 20 signaling pathways in the results, were selected for visual analysis. Additionally, column and bubble plots were generated to illustrate the results of the GO and KEGG analyses.

### Construction of drug-ingredient-target network

To explore the therapeutic mechanisms of RB and HMM in liver fibrosis, the active ingredients and hub targets were loaded into Cytoscape 3.9.1 to construct a drug-target-disease network. The top 10 active ingredients, based on their Degree ranking, were selected for visualization.

### Microarray data analysis

The microarray datasets were downloaded from the Gene Expression Omnibus-NCBI (GEO) database (a comprehensive biotechnology information and gene expression database https://www.ncbi.nlm.nih.gov/geo) by a keyword such as “liver fibrosis” and the species was defined as Homo sapiens [26]. Later, a package of R language “Limma” (an effective tool for reading, normalizing, and interpreting gene expression data) was utilized for the screening of differentially expressed genes (DEGs). We used the tool GEO2R (http://www.ncbi.nlm.nih.gov/geo/geo2r/) to analyze the DEGs in the liver fibrosis group and the control group. Screening conditions were set as p val <0.05 and |log (FC)| ≥ 0.5, |log(FC)| < −0.5. The DEGs were compared with the hub genes, and the genes common to both sets were selected for further analysis, including molecular docking and dynamic simulation.

### Survival analysis and pathological staging

The survival analysis and pathological staging of final hub genes were constructed utilizing GEPIA2 (http://gepia2.cancer-pku.cn/), which is an enhanced web server for large-scale expression profiling and interactive analysis [27]. Within the GEPIA2 platform, Kaplan-Meier survival curves and box plots representing liver cancer stages were generated using data related to liver fibrosis, in order to explore the relationship between the hub genes and both overall survival and pathological staging in liver fibrosis patients.

### Molecular docking studies

Molecular docking was performed using the Glide module in Schrodinger 2017 to explore the interaction modes between three common components (MOL000098, MOL000354, MOL000422) and four final hub proteins (TNF, IL-6, PPARG, MMP9), respectively [28]. The crystal structures of TNF (PDB ID: 6X81), IL-6 (PDB ID: 5SFK), PPARG (PDB ID: 6TSG) and MMP9 (PDB ID: 8K5Y) were all downloaded from the Protein Data Bank (www.rcsb.org) [29–32]. The method of docking was referred to in our previous article [33]. Ultimately, suitable docking models were selected from the diverse clusters formed between the ligands and receptors, focusing on those with lower binding energies for subsequent molecular dynamics simulation analysis. The intermolecular interactions were visualized using PyMOL software [34].

### Molecular dynamics simulation

Molecular dynamics simulations (MD) of different systems were analyzed using Amber 14 software for 50 ns simulation based on the reasonable conformation complex obtained by molecular docking [35]. The general procedure was from our previous article [36]. Finally, important data such as the root mean square deviations (RMSD), root mean square fluctuation (RMSF), binding free energy and decomposition free energy by the MM_GBSA and MM_PBSA methods, and intermolecular interactions, were obtained.

### Cell culture and proliferation assay

Human hepatic cell line L02 and human hepatic stellate cell line LX2 were obtained from the Type Culture Collection of the Chinese Academy of Sciences (Shanghai, China), and were propagated in DMEM containing 10% FBS, 100mM penicillin and 100mM streptomycin at 37°C in a humidified atmosphere containing 5% CO_2_.

Cell proliferation assay was performed using MTT assay in a 96-well plate [23]. L02 cell line (5000 cells per well) was treated with MOL000098 (quercetin), MOL000354 (isorhamnetin) and MOL000422 (kaempferol) at different concentrations (10, 20, 40 μM) for 2 h and then incubated with 20 ng/ml TGFβ for another 72 h, while LX2 cells were treated various concentrations (1.25, 2.5, 5, 10, 20, 40 μM) of the three compounds. Then 20 μL, 4 mg/mL MTT of PBS solution was added to each well for 4 h. The medium was carefully removed, and the formazan crystals were dissolved in 200 μL of DMSO in the dark after which absorbance values at 490 nm wavelength were read on a Super Microplate Reader (MQX2OO) (BioTek, the United States).

### Western blotting

Antibodies against TNF-α (D5G9), IL-6 (D3K2N) and β-actin (13E5) were purchased from Cell Signaling Technology. Briefly, the quantified protein samples were subjected to an SDS-PAGE and then transferred to a PVDF membrane. After incubating with primary antibodies at 4°C overnight and secondary antibodies at room temperature for 2 h, the membrane was illuminated with ECL solution, and the density was analyzed using Image J.

## Results and discussion

### Prediction of potential targets for compounds and disease

Based on the TCMSP and Swiss ADME platform, a total of 17 effective ingredients of RB and 20 of HMM were obtained, among which common components were MOL000098 (quercetin), MOL000354 (isorhamnetin) and MOL000422 (kaempferol) (Table 1). Meanwhile, 601 targets interacting with those putative active ingredients were obtained from the Swiss Target Prediction whose probability is *>*0.10 in the prediction results for further analysis. In addition, a total of 1661 target genes against liver fibrosis were also screened by filtering the metrics of median value through GeneCard and OMIM databases.

**Table 1:** The active ingredients in RB-HMM.

Drug	Mol ID	Molecule Name	OB (%)	DL
RB	MOL001645	Linoleyl acetate	42.1	0.2
	MOL002776	Baicalin	40.12	0.75
	MOL000449	Stigmasterol	43.83	0.76
	MOL004598	3,5,6,7-tetramethoxy-2-(3,4,5-trimethoxyphenyl)chromone	31.97	0.59
	MOL004609	Areapillin	48.96	0.41
	MOL013187	Cubebin	57.13	0.64
	MOL004624	Longikaurin A	47.72	0.53
	MOL004628	Octalupine	47.82	0.28
	MOL004644	Sainfuran	79.91	0.23
	MOL004648	Troxerutin	31.6	0.28
	MOL004653	(+)-Anomalin	46.06	0.66
	MOL004702	saikosaponin c_qt	30.5	0.63
	MOL004718	α-spinasterol	42.98	0.76
	MOL000490	petunidin	30.05	0.31
Common ingredients	MOL000354	isorhamnetin	49.6	0.31
	MOL000422	kaempferol	41.88	0.24
	MOL000098	quercetin	46.43	0.28
HMM	MOL000211	Mairin	55.38	0.78
	MOL000239	Jaranol	50.83	0.29
	MOL000296	hederagenin	36.91	0.75
	MOL000033	(3S,8S,9S,10R,13R,14S,17R)-10,13-dimethyl-17-[(2R,5S)-5-propan-2-yloctan-2-yl]-2,3,4,7,8,9,11,12,14,15,16,17-dodecahydro-1H-cyclopenta[a]phenanthren-3-ol	36.23	0.78
	MOL000371	3,9-di-O-methylnissolin	53.74	0.48
	MOL000374	5’-hydroxyiso-muronulatol-2’,5’-di-O-glucoside	41.72	0.69
	MOL000378	7-O-methylisomucronulatol	74.69	0.3
	MOL000379	9,10-dimethoxypterocarpan-3-O-β-D-glucoside	36.74	0.92
	MOL000380	(6aR,11aR)-9,10-dimethoxy-6a,11a-dihydro-6H-benzofurano[3,2-c]chromen-3-ol	64.26	0.42
	MOL000387	Bifendate	31.1	0.67
	MOL000392	formononetin	69.67	0.21
	MOL000398	isoflavanone	109.99	0.3
	MOL000417	Calycosin	47.75	0.24
	MOL000433	FA	68.96	0.71
	MOL000438	(3R)-3-(2-hydroxy-3,4-dimethoxyphenyl)chroman-7-ol	67.67	0.26
	MOL000439	isomucronulatol-7,2’-di-O-glucosiole	49.28	0.62
	MOL000442	1,7-Dihydroxy-3,9-dimethoxy pterocarpene	39.05	0.48

### Establishment of PPI network analysis

**Fig 2:**
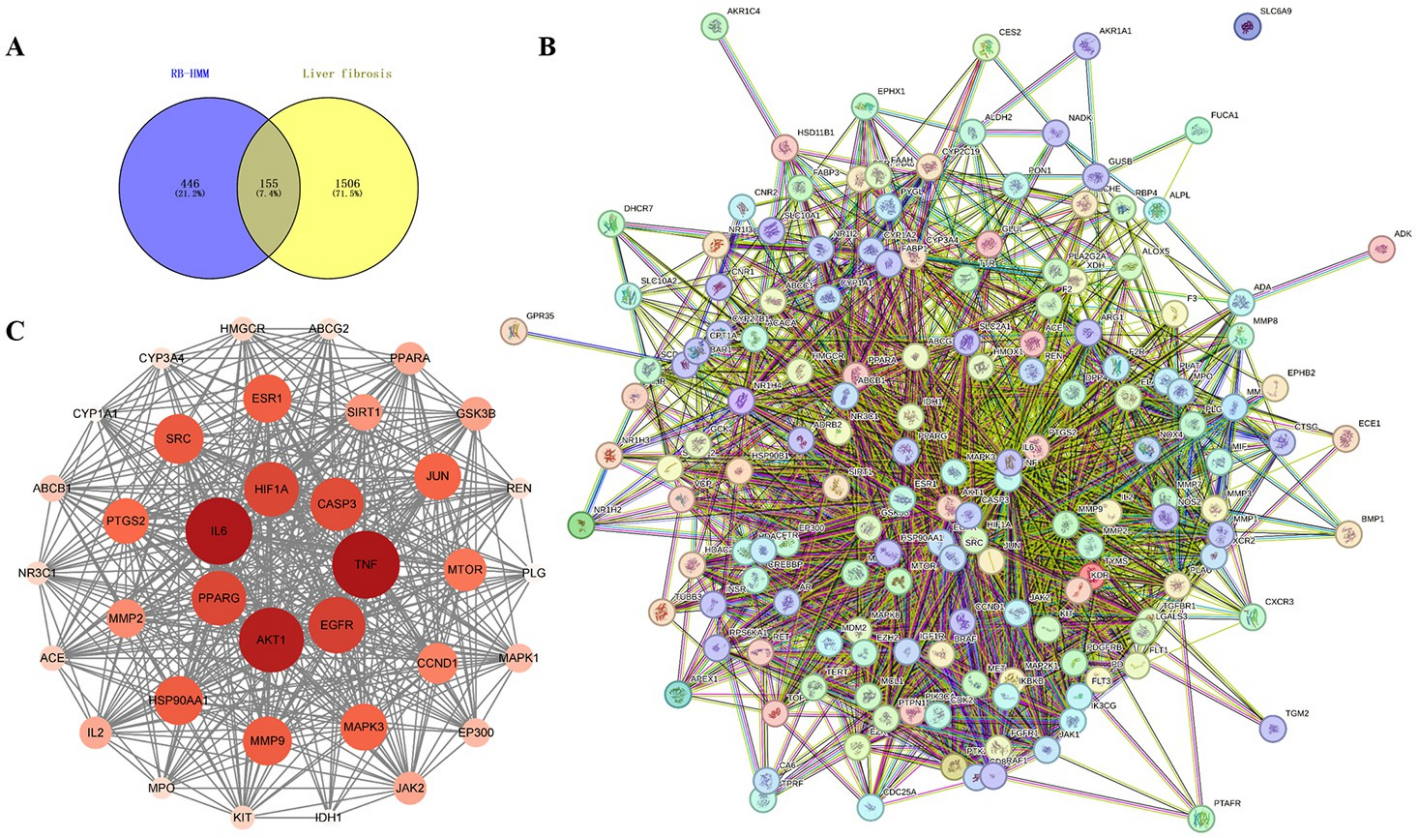
(A) The Venn diagram of the common genes between compounds (RB-HMM) and disease (liver fibrosis), (B) PPI network analysis, (C) The 36 hub genes.

To screen the critical targets, the result of the Venn diagram (Fig 2A) was established through Venny 2.1, which suggested that 155 overlapping genes were obtained by intersecting 601 compound-related genes with 1661 disease-related genes for further mechanisms study of RB and HMM on the treatment of liver fibrosis. As shown in Fig 2B, the protein relationship was predicted through the online databases and Cytoscape 3.9.1 software. A PPI network consisting of 155 nodes and 2433 edges with an average connectivity of 31.4 was obtained on the String platform. Subsequently, the PPI network was imported into Cytoscape 3.9.1 for visualization and analysis. As shown in Fig 2C, 36 hub target genes were ultimately identified by median screen using three algorithms, Degree (median: 31.5974025974026), Closeness (median: 0.0034487176713530266), and Betweenness (median: 143.5584415584406). Meanwhile, the species types were homo sapiens and the other parameters were default. The darker the color and larger the area of the circular node, the more important the node is in the network. These findings suggested that multiple components and multiple targets may contribute to the improving effect of RB and HMM on the treatment of liver fibrosis and the top 10 hub targets TNF, IL-6, AKT1, EGFR, HIF1A, PPARG, CASP3, SRC, MMP9 and HSP90AA1 (Table 2) were regarded as targets for direct interaction between compounds and diseases.

**Table 2:** The top 10 hub targets.

Rank	Name	Degree	Closeness	Betweenness
1	TNF	107	0.005025126	301.1164339
2	IL-6	106	0.004975124	301.1164339
3	AKT1	103	0.004807692	301.1164339
4	EGFR	91	0.00456621	301.1164339
5	PPARG	88	0.004545455	301.1164339
6	HIF1A	88	0.004464286	301.1164339
7	CASP3	86	0.004444444	301.1164339
8	SRC	81	0.004347826	301.1164339
9	MMP9	80	0.004310345	301.1164339
10	HSP90AA1	80	0.004329004	301.1164339

### GO and KEGG analysis

**Fig 3:**
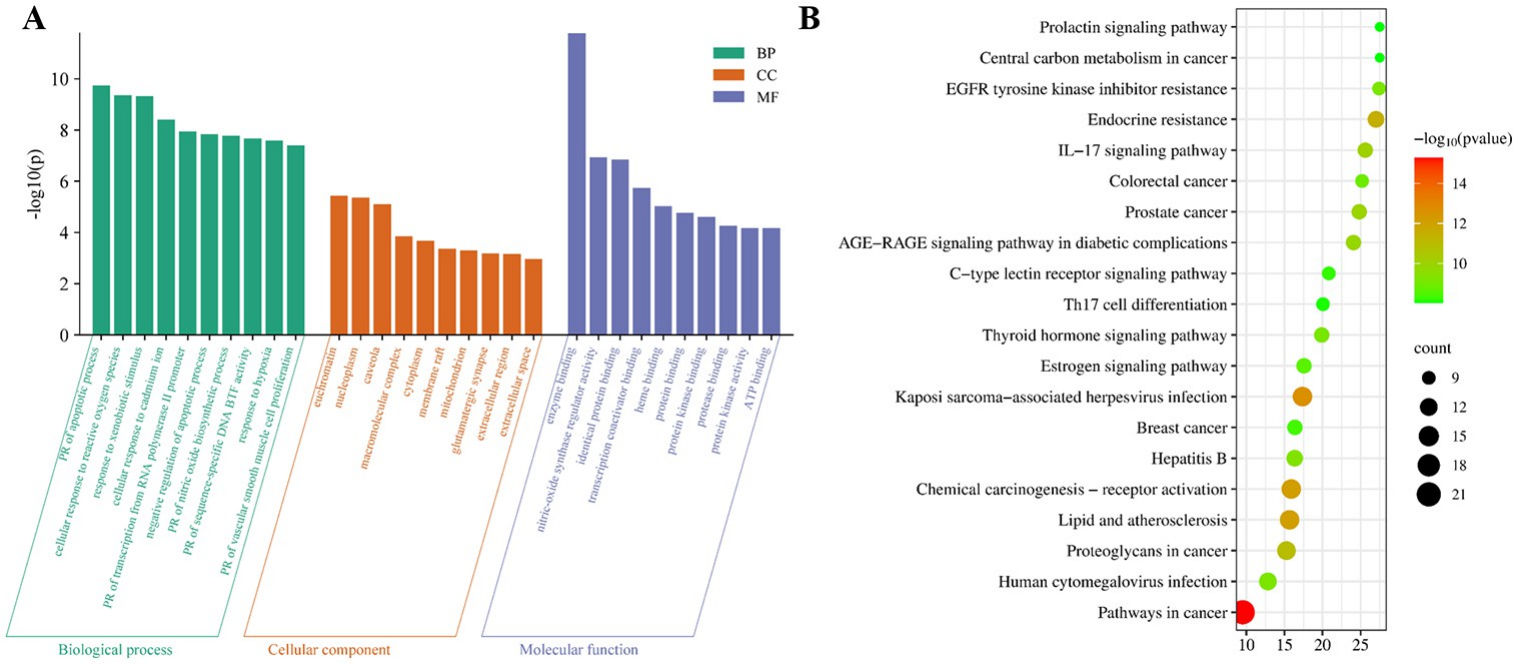
(A) GO enrichment analysis of therapeutic targets (the top 10 results of BP, CC, and MF enrichment analysis respectively), (B) KEGG enrichment analysis of therapeutic targets (the top 20 results). PR was the abbreviation of positive regulation, and BTF was the abbreviation of the binding transcription factor.

The online David database was used to conduct GO and KEGG enrichment analysis on the potential targets of RB and HMM for liver fibrosis, and P < 0.05 was used as the evaluation criterion. Screening results (Fig 3) showed that 335 biological processes (BP), 39 cellular components (CC), 78 molecular functions (MF), and 139 signaling pathways in the results were enriched. The top 10 biological functions and top 20 signaling pathways were visually analyzed. The bar charts and bubble charts analyzed by GO and KEGG were finally obtained and illustrated using the website (http://www.bioinformatics.com.cn/). BP mainly included PR of apoptotic process (GO:0043065), cellular response to reactive oxygen species (GO:0034614), response to xenobiotic stimulus (GO:0009410), cellular response to cadmium ion (GO:0071276), PR of transcription from RNA polymerase II promoter (GO:0045944), negative regulation of apoptotic process (GO:0043066), PR of nitric oxide biosynthetic process (GO:0045429), PR of sequence-specific DNA BTF activity (GO:0051091), response to hypoxia (GO:0001666), PR of vascular smooth muscle cell proliferation (GO:1904707). CC enrichment mainly focused on euchromatin (GO:0000791), nucleoplasm (GO:0005654), caveola (GO:0005901), macromolecular complex (GO:0032991), cytoplasm (GO:0005737), membrane raft (GO:0045121), mitochondrion (GO:0005739), glutamatergic synapse (GO:0098978), extracellular region (GO:0005576), extracellular space (GO:0005615). MF mainly included enzyme binding (GO:0019899), nitric-oxide synthase regulator activity (GO:0030235), identical protein binding (GO:0042802), transcription coactivator binding (GO:0001223), heme binding (GO:0020037), protein binding (GO:0005515), protein kinase binding (GO:0019901), protease binding (GO:0002020), protein kinase activity (GO:0004672), ATP binding (GO:0005524). KEGG pathway enrichment analysis showed that the main signaling pathways were pathways in cancer, kaposi sarcoma-associated herpesvirus infection chemical carcinogenesis-receptor activation, lipid and atherosclerosis, endocrine resistance, proteoglycans in cancer, IL-17 signaling pathway, prostate cancer, AGE-RAGE signaling pathway in diabetic complications, hepatitis B, EGFR tyrosine kinase inhibitor resistance, human cytomegalovirus infection, thyroid hormone signaling pathway, colorectal cancer, estrogen signaling pathway, breast cancer, C-type lectin receptor signaling pathway, Th17 cell differentiation, central carbon metabolism in cancer, prolactin signaling pathway. These results showed that the molecular mechanism may be related to regulating the cell process, such as cell apoptotic through protein binding function and immunoinflammatory response pathway.

### Construction of network

**Fig 4:**
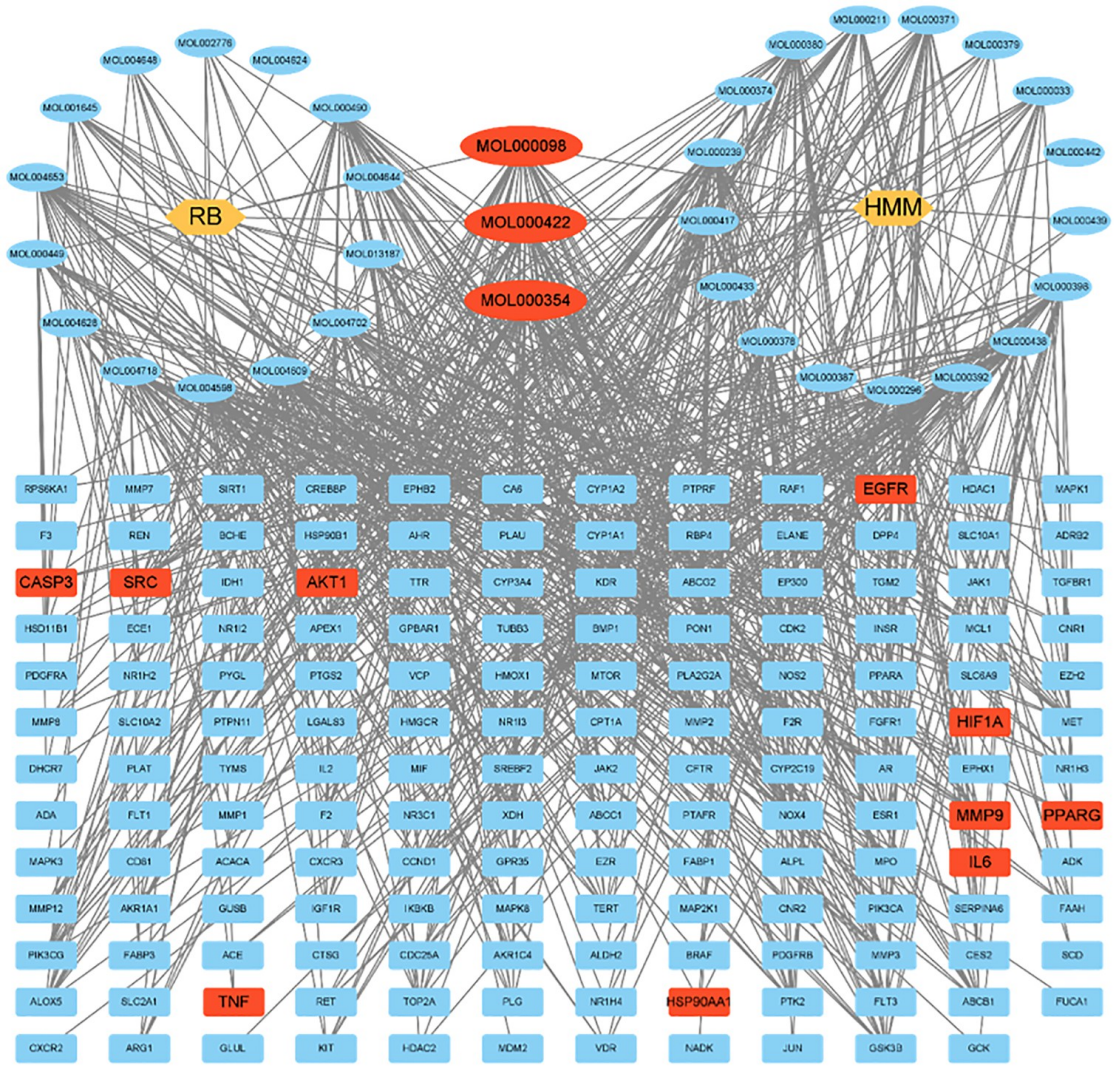
The network of the relationship between the active ingredients of RB-HMM and the targets of liver fibrosis. The three common components (MOL000098, MOL000354, MOL000422) and the top 10 hub targets were colored red.

As shown in Fig 4, the 34 active ingredients and 155 common targets were imported into Cytoscape 3.9.1 to construct the “drug-ingredient-target” network. According to Degree ranking, the top 10 active ingredients in Table 3 were selected as key active ingredients in anti-liver fibrosis activity and associated with multiple target genes. Among them, the number of associated target genes of three common components (MOL000098, MOL000354, MOL000422) were 39, 37 and 36, respectively, all of which belonged to flavonoid active molecules and had significant inhibitory effects on anti-fibrosis and anti-tumor, especially in the field of liver fibrosis and HCC (liver hepatocellular carcinoma). Moreover, the three active molecules were highly correlated with the nodes in the innermost circle of 36 hub targets. The GO functional annotation and KEGG-related pathway enrichment analysis showed that immune system-related inflammatory factors such as TNF, IL-6, DNA bind to transcription factors such as PPARG, and cell apoptosis regulators such as MMP9, played important roles in liver fibrosis progression.

**Table 3:** The top 10 active ingredients.

Rank	Degree	TCMSP ID	Name	Drug
1	39	MOL000354	Isorhamnetin	Common
2	39	MOL004598	3,5,6,7-tetramethoxy-2-(3,4,5-trimethoxyphenyl) chromone	RB
3	39	MOL000239	Jaranol	HMM
4	38	MOL004609	Areapillin	RB
5	38	MOL000490	Petunidin	RB
6	37	MOL000098	Quercetin	Common
7	36	MOL000422	Kaempferol	Common
8	35	MOL000438	(3R)-3-(2-hydroxy-3,4-dimethoxyphenyl)chroman-7-ol	HMM
9	33	MOL000380	(6aR,11aR)-9,10-dimethoxy-6a,11a-dihydro-6H-benzofurano[3,2-c]chromen-3-ol	HMM
10	32	MOL000398	Isoflavanone	HMM

### Microarray data analysis

The four microarray data GSE139994, GSE84044, GSE49541 and GSE163211 related to liver fibrosis were downloaded from the GEO database. The GSE139994 (platform GPL1355) microarray data contained three vehicle treatment liver tissue samples from normal rats and three liver fibrosis tissue samples from CCl_4_ treatment liver fibrosis rats. The GSE84044 (platform GPL570) microarray data contained 43 hepatic tissue from patients with chronic hepatitis B (CHB) with liver fibrosis and inflammation stage 0 and 28 samples from patients with CHB with liver fibrosis stage 3 to 4. The GSE49541 (platform GPL570) microarray data contained 40 NAFLD liver biopsy tissue with mild fibrosis stage 0 to 1 and 32 samples with advanced fibrosis stage 3 to 4. The GSE163211 (platform GPL29503) contained 82 liver fibrosis samples and 76 normal samples of human liver tissues.

The DEGs were then identified using the R package “Limma”. Only those genes were considered as DEGs which retained the criteria of p val <0.05 and |log (FC)| ≥ 0.5, |log(FC)| < −0.5. From the top 10 hub genes, TNF and IL-6 were found in all four datasets (GSE139994, GSE84044, GSE49541 and GSE163211). PPARG and MMP9 were found in three datasets (GSE139994, GSE84044 and GSE163211). TNF and MMP9 were downregulated while IL-6 and PPARG were upregulated in all four datasets. These four genes named TNF, IL-6, PPARG and MMP9 were processed further for survival analysis and pathological staging.

### Pathological staging and survival analysis

**Fig 5:**
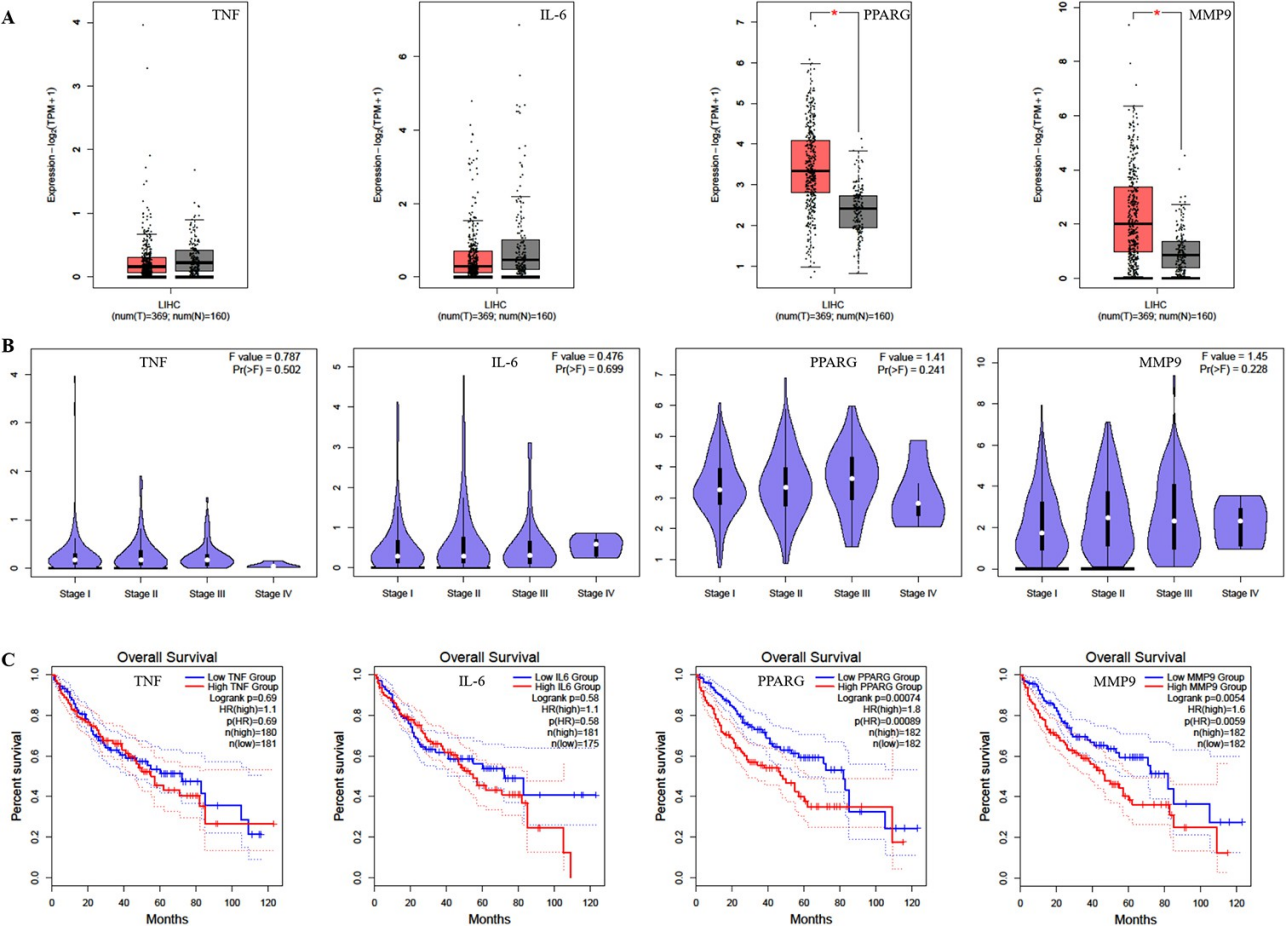
The expressions and survival analysis of TNF, IL-6, PPARG and MMP9 in patients with HCC. (A) The expressions of TNF, IL-6, PPARG and MMP9 in HCC using box plots. (B) The expressions of TNF, IL-6, PPARG and MMP9 in different stages of HCC using violin plots. (C) Survival analysis of TNF, IL-6, PPARG and MMP9.

Patients with progressive liver fibrosis are more likely to develop liver cancer, especially HCC [37]. The clinical importance of these four genes named TNF, IL-6, PPARG and MMP9 were analyzed using survival analysis and pathological staging based on gene expression levels. The results in Fig 5A revealed that the expressions of PPARG and MMP9 in 369 tumor tissues were significantly higher than those in 160 normal tissues in HCC, while the expressions of TNF and IL-6 were lower than those in normal tissues. The expressions of TNF, PPARG and MMP9 were negatively correlated with the pathological stage of the tumor from stage I to IV in Fig 5B. As shown in Fig 5C, the overall survival of patients with HCC with high expressions of IL-6, PPARG and MMP9 was significantly shorter than that of patients with low expressions of these genes. On the contrary, the overall survival of patients with high expression of TNF in tumor tissues was significantly longer than that of patients with low expressions of this gene. Therefore, TNF, IL-6, PPARG and MMP9 might be the potential biomarkers and therapeutic targets related to the progression of liver fibrosis to HCC.

### Molecular docking analysis

To explore the specific binding mode of three common active ingredients (MOL000098, MOL000354, MOL000422) in the top 10 with final four hub targets (TNF, IL-6, PPARG, MMP9), molecular docking was conducted in this study by the Glide module in Schrodinger 2017. The crystal structures of TNF (PDB ID: 6X81, resolution: 2.81 Å, R_free_: 0.231), IL-6 (PDB ID: 5SFK, resolution: 2.33 Å, R_free_: 0.248), PPARG (PDB ID: 6TSG, resolution: 2.98 Å, R_free_: 0.292) and MMP9 (PDB ID: 8K5Y, resolution: 1.52 Å, R_free_: 0.198) were downloaded from the Protein Data Bank (www.rcsb.org). The three common active ingredients (MOL000098, MOL000354, MOL000422) and endogenous ligands were docked by standard precision (SP) glide and flexible dock according to the protocols. As shown in Table 4, the docking scores of three common active ingredients (MOL000098, MOL000354, MOL000422) to final four hub targets (TNF, IL-6, PPARG, MMP9), respectively, were stronger than four endogenous ligands, which was conducive to playing a pivotal role in protein binding affinity and stability using the SP method.

**Table 4:** Docking results of three common active ingredients (MOL000098, MOL000354, MOL000422) with final four hub targets (TNF, IL-6, PPARG, MMP9) using SP model.

Proteins	Molecules	Docking scores (Kcal/mol)	Key residues
TNF(6X81)	MOL000398	-9.664	GLN61, LEU120(H-bond), TYR119(Pi-Pi stacking)
	MOL000354	-9.371	SER60, TYR151(H-bond), TYR119(Pi-Pi stacking)
	MOL004609	-9.057	LEU120, TYR151(H-bond), TYR119(Pi-Pi stacking)
	MOL000239	-9.045	TYR151(H-bond), TYR119(Pi-Pi stacking)
	MOL000438	-9.023	GLN61, TYR151(H-bond)
	MOL000098	-8.969	TYR151(H-bond), TYR119(Pi-Pi stacking)
	MOL000380	-8.795	TYR151(H-bond), TYR119(Pi-Pi stacking)
	MOL000422	-8.638	GLN61, TYR151(H-bond), TYR119(Pi-Pi stacking)
	MOL004598	-7.338	TYR151(H-bond)
	MOL000490	-7.155	LEU120(H-bond)
	6X81_ligand	-7.672	TYR59(Pi-Pi stacking)
IL-6(5SFK)	MOL000098	-9.514	THR685, GLN726(H-bond), PHE729(Pi-Pi stacking)
	MOL000422	-9.437	THR685(H-bond), PHE729(Pi-Pi stacking)
	MOL000239	-8.867	THR685, GLN726(H-bond), PHE729(Pi-Pi stacking)
	MOL000354	-8.807	THR685, GLN726(H-bond), PHE729(Pi-Pi stacking)
	MOL004609	-8.149	THR685(H-bond), PHE696, PHE729(Pi-Pi stacking)
	MOL000380	-8.06	PHE696(Pi-Pi stacking)
	MOL000438	-7.638	GLN726(H-bond)
	MOL004598	-7.125	GLN726(H-bond), PHE696, PHE729(Pi-Pi stacking)
	MOL000398	-7.005	GLU592, ASP674(H-bond), PHE696, PHE729(Pi-Pi stacking)
	MOL000490	-6.941	THR633, GLN726(H-bond), HIP525(Pi-cation)
	5SFK_ligand	-7.208	ASP564, GLU592(H-bond)
PPARG(6TSG)	MOL000398	-8.263	LEU228, ARG288, GLU295(H-bond)
	MOL000438	-7.847	ARG288, SER289, TYR327(H-bond)
	MOL000098	-7.618	ARG288, SER289(H-bond)
	MOL000239	-7.403	ARG288(H-bond)
	MOL000354	-7.011	ARG288(H-bond)
	MOL000422	-6.925	ARG288(H-bond)
	MOL000380	-6.902	SER289(H-bond)
	MOL004609	-6.793	ARG288, TYR327(H-bond)
	MOL004598	-6.596	ARG288(H-bond)
	MOL000490	-6.573	ARG288, SER289(H-bond)
	6TSG_ligand	-6.452	SER289, TYR327(H-bond)
MMP9(8K5Y)	MOL000438	-9.515	GLN108, GLY233(H-bond)
	MOL000422	-8.134	ARG106, PRO180, ALA191(H-bond)
	MOL000098	-7.841	ARG106, PRO180, ALA191(H-bond)
	MOL000354	-7.668	ARG51, ARG106, GLN108(H-bond), TYR179, HIE190(Pi-Pi stacking)
	MOL000380	-7.639	ARG106, PRO180(H-bond)
	MOL000398	-7.608	ARG106, GLN108, ALA191(H-bond), PHE110, TYR179(Pi-Pi stacking)
	MOL004609	-7.531	ARG106, GLN108, PRO180(H-bond), TYR179(Pi-Pi stacking)
	MOL000239	-7.468	ARG51, ALA191(H-bond)
	MOL000490	-6.901	ARG51, ARG106, GLN108, HIE190(H-bond)
	MOL004598	-6.654	ARG51, ARG106, GLN108(H-bond), TYR179(Pi-Pi stacking)
	8K5Y_ligand	-7.159	ARG51, ARG106, ALA191(H-bond)

**Fig 6:**
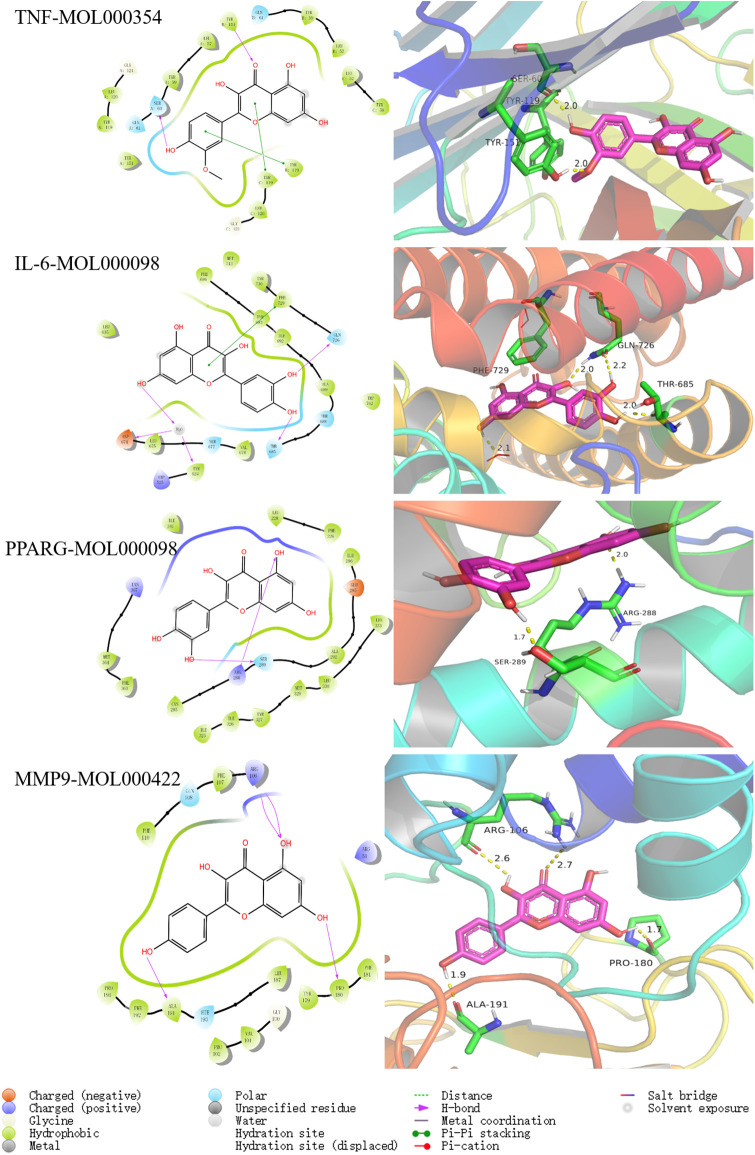
The interaction patterns of the most stable systems in each protein (TNF-MOL000354, IL-6-MOL000098, PPARG-MOL000098 and MMP9-MOL000422) showed the electrostatic surface in the 2D and 3D representation from molecular docking. The molecules (red) and key residues (green) were represented in sticks and coloured by atom type.

We found that the binding energies of all molecular docking results were less than -6.5, which indicated a more vigorous binding activity [38]. Among them, three common active ingredients named MOL000098, MOL000354, and MOL000422 were strongly bound with TNF protein with a binding affinity of -9.371, -8.969, and -8.638 kJ/mol, respectively. All compounds were bound to key residues GLN61, TYR151 and LEU120 of TNF through intermolecular hydrogen bonds and formed Pi-Pi stacking with key residue TYR119. Remarkably, MOL000098 exhibited the highest affinity (-9.524 kJ/mol) with IL-6 protein among all of the molecules, and MOL000422 (-9.437 kJ/mol), MOL000354 (-8.807 kJ/mol) also showed stronger binding affinity. The side chains of the top 10 active ingredients formed hydrogen bonds with THR685 and GLN726 and formed Pi-Pi stacking with PHE729. The docking scores of MOL000098, MOL000354 and MOL000422 binding to PPARG protein were -7.618, -7.011, and -6.925 kJ/mol, respectively. The intermolecular forces H-bond mainly consisted of ARG288, SER289 and TYR327. In addition, MOL000422, MOL000098 and MOL000354 displayed stably bound with MMP9 protein with binding energies of -8.134, -7.841, and -7.668 kJ/mol, respectively. The H-bonds with key residues such as ARG51, ARG106, GLN108, PRO180 and ALA191, and Pi-Pi stacking with TYR179 exerted significant effects on anti-liver fibrosis activity. The interaction patterns of the most stable systems in each protein (TNF-MOL000354, IL-6-MOL000098, PPARG-MOL000098 and MMP9-MOL000422) were illustrated in Fig 6. These results of molecular docking may contribute to the difference in their effect activity.

### Molecular dynamics simulation

Molecular dynamics simulation analysis of 12 systems (TNF-MOL000098, TNF-MOL000354, TNF-MOL000422, IL-6-MOL000098, IL-6-MOL000354, IL-6-MOL000422, PPARG-MOL000098, PPARG-MOL000354, PPARG-MOL000422, MMP9-MOL000098, MMP9-MOL000354, MMP9-MOL000422) were performed for 50 ns by Amber 14 software.

**Fig 7:**
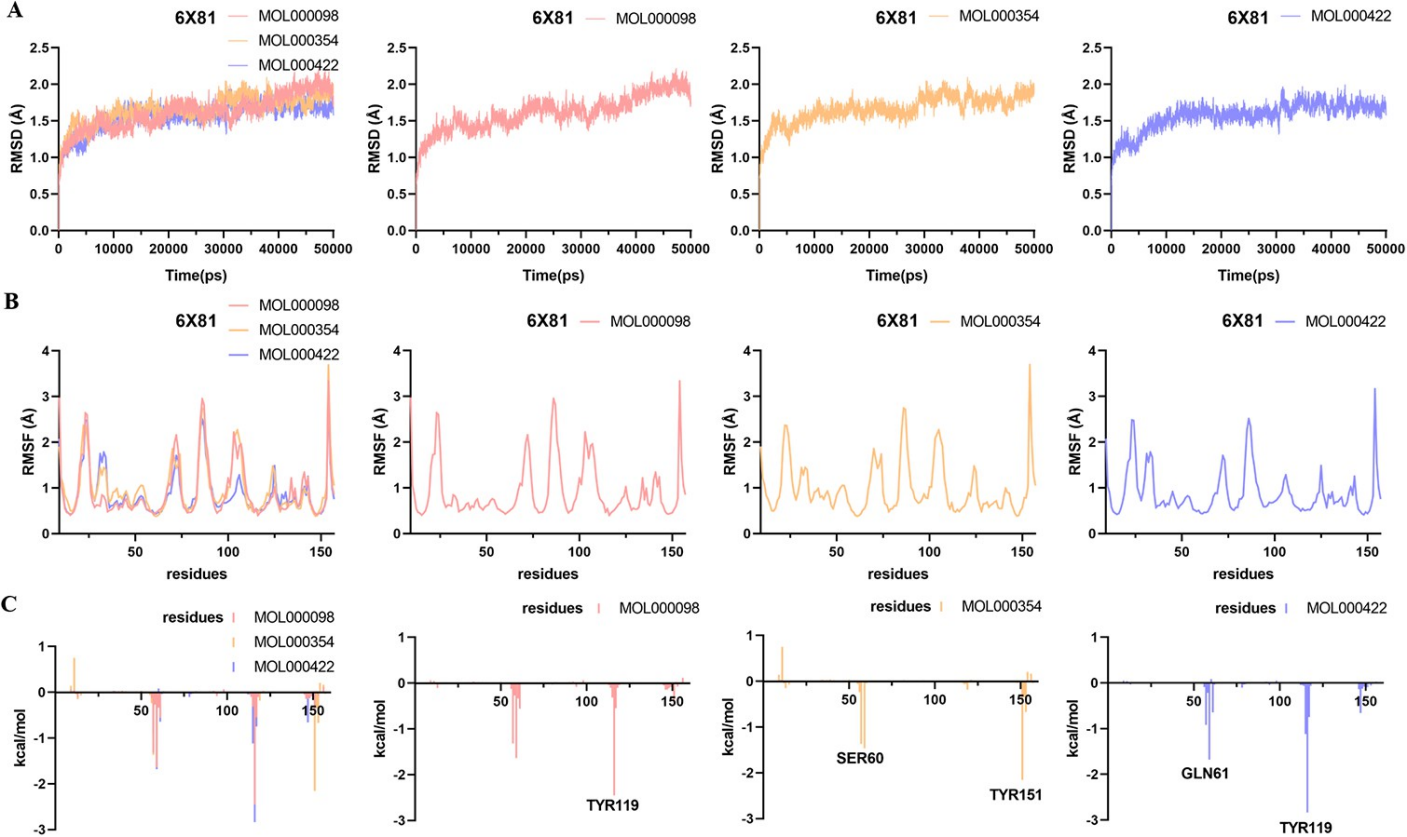
Molecular dynamics simulations of the three systems (TNF-MOL000098, TNF-MOL000354, TNF-MOL000422) after 50 ns. (A) RMSD change, (B) RMSF change and (C) Decomposition free energy.

**Fig 8:**
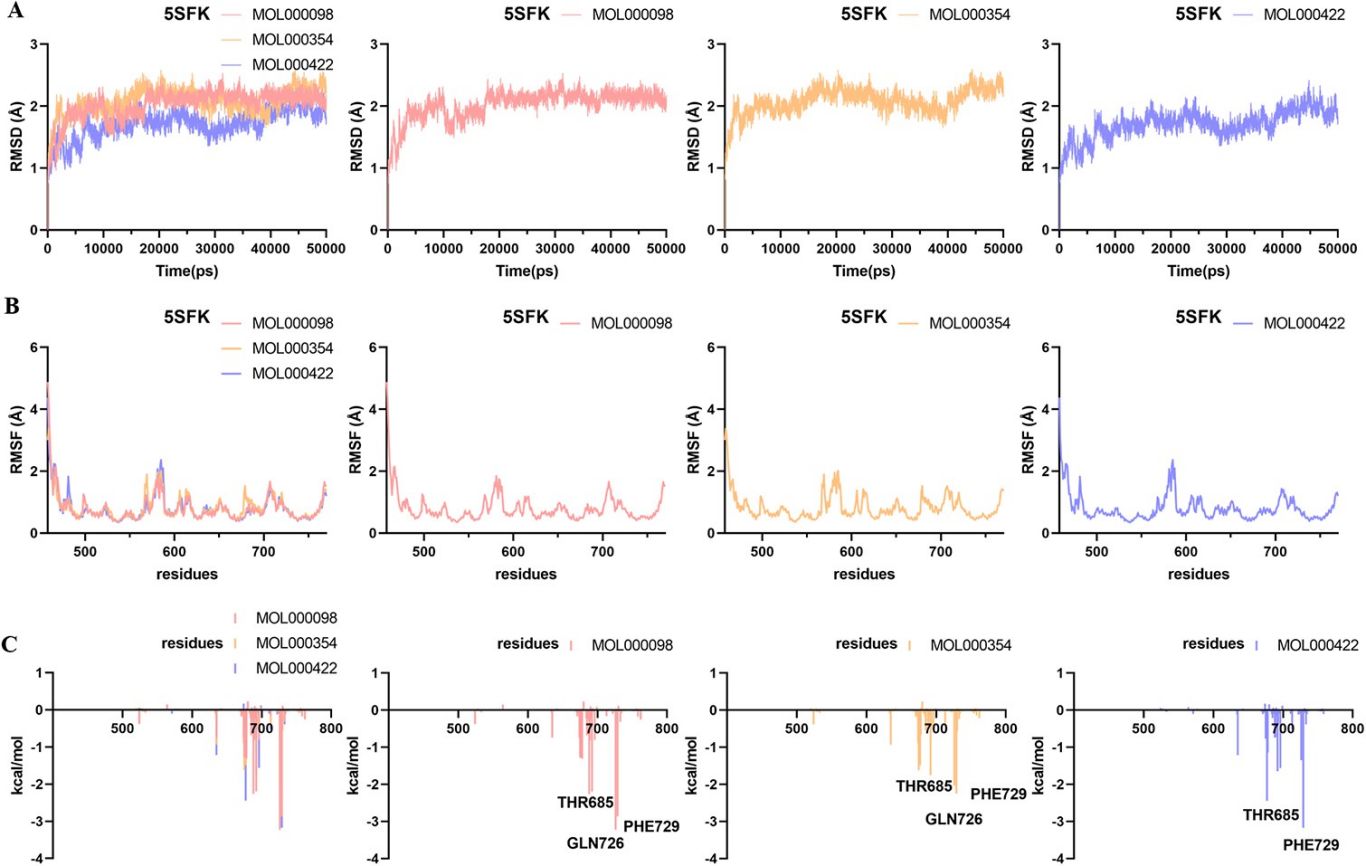
Molecular dynamics simulations of the three systems (IL-6-MOL000098, IL-6-MOL000354, IL-6-MOL000422) after 50 ns. (A) RMSD change, (B) RMSF change and (C) Decomposition free energy.

**Fig 9:**
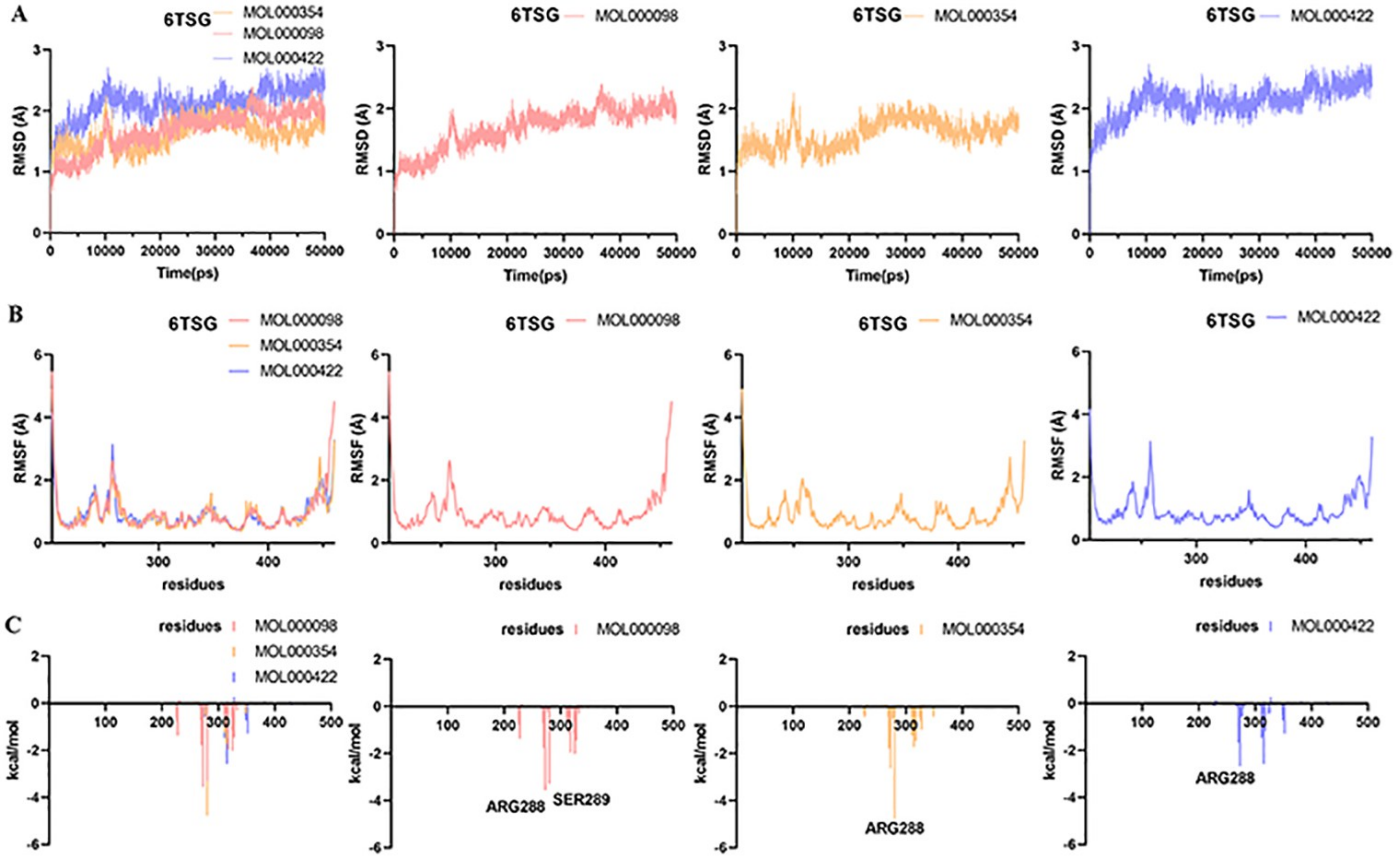
Molecular dynamics simulations of the three systems (PPARG-MOL000098, PPARG-MOL000354, PPARG-MOL000422) after 50 ns. (A) RMSD change, (B) RMSF change and (C) Decomposition free energy.

**Fig 10:**
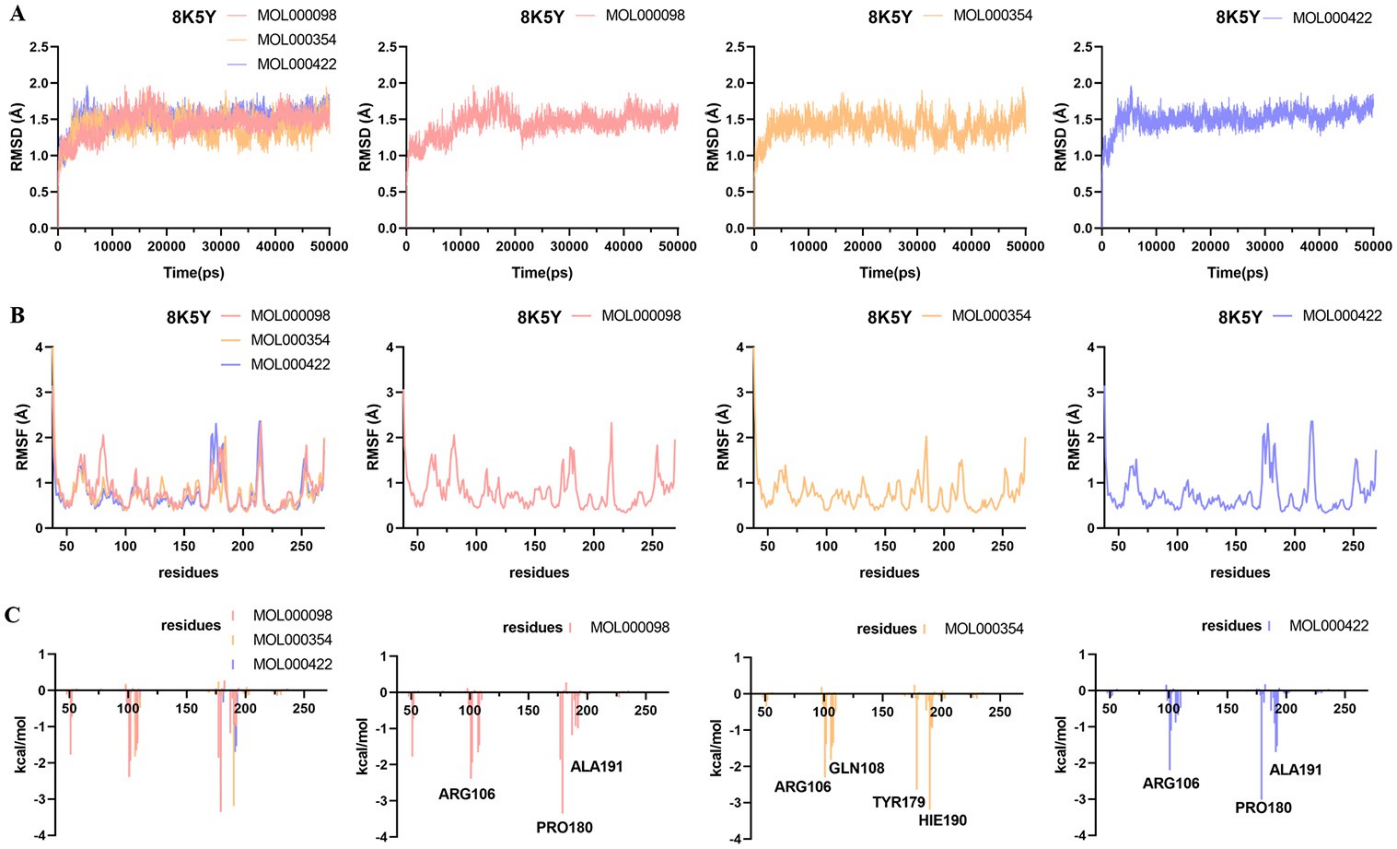
Molecular dynamics simulations of the three systems (MMP9-MOL000098, MMP9-MOL000354, MMP9-MOL000422) after 50 ns. (A) RMSD change, (B) RMSF change and (C) Decomposition free energy.

The RMSD [39] is considered as a measure of system stability. The lower the mean RMSD value, the more stable the body system. As shown in Figs 7A–10A, the 12 systems reached a state of equilibrium and convergence throughout the simulated 50 ns process, which was stable at about 1.7 Å (TNF), 2 Å (IL-6), 2 Å (PPARG) and 1.5 Å (MMP9), respectively. The RMSD showed that the ligand and receptor bind closely and the complex was stable, indicating that the simulation results can be further analyzed.

The RMSF [40] can represent the fluctuation of small molecular ligands on the spatial structure of proteins. The lower the value, the more stable the residue of the protein interacts with the small molecule. According to the observation in Figs 7B–10B, the RMSF exhibited that the amino acid fluctuations of each three systems were significantly similar, suggesting that these residues with lower RMSF values may be key residues.

To find out the contribution value of active site residues, we calculated the decomposition-free energy of these 12 systems by the MM_GBSA and MM_PBSA methods. As shown in Figs 7C–10C, amino acids with higher contribution values and low RMSF values in the 12 systems were consistent with the results of molecular docking, which may be due to strong interaction forces in the systems. As shown in Fig 7C, the molecules MOL000098, MOL000354, MOL000422 interacted with the key residues TYR119, SER60 and TYR151, GLN61 and TYR119 in TNF (PDB ID: 6X81) protein with energy values -2.45, -1.56 and -2.15, -1.68 and -2.84 kcal/mol, respectively. Similarly, the contributions of energy were all lower than -1.5 kcal/mol in the remaining nine systems, these were IL-6 (PDB ID: 5SFK), PPARG (PDB ID: 6TSG) and MMP9 (PDB ID: 8K5Y) proteins (Figs 8–10).

To quantify the interaction of 12 systems, the binding free energy was also calculated based on van der Waals force energy, electrostatic energy, polar solvent energy, and non-polar solvent energy using the MM_GBSA and MM_PBSA methods. In general, the last 2 ns were selected from the equilibrium phase of the trajectory to calculate the binding free energy by the following three formulas [36].

After 50 ns dynamics simulation, the contributions of each energy term were listed in Table 5 by MM-GBSA method and Table 6 by MM-PBSA method. The total binding free energy (ΔG_total_) of 12 systems were all lower than -20 kcal/mol by the MM-GBSA method and -10 kcal/mol by the MM-PBSA method, indicating that the 12 systems were stable. Remarkably, the PPARG-MOL000098 system was the most stable among the 12 systems with a ΔG_total_ of -33.41 by the MM-GBSA method and -19.88 kcal/mol by the MM-PBSA method. The van der Waals interactions (for example in the TNF-MOL000098 system, E_vdw_ = -37.53 ± 0.70 kcal/mol) played a more important role than the electrostatic interaction (E_el_ = -23.31 ± 1.42 kcal/mol). In addition, the polar interactions (E_el_+E_gb_) of these 12 systems were greater than the nonpolar interactions (E_vdw_+E_surf_), suggesting that the nonpolar interactions in the 12 systems, especially E_vdw_, were more favorable for the binding of the molecules MOL000098, MOL000354, MOL000422 to TNF (PDB ID: 6X81), IL-6 (PDB ID: 5SFK), PPARG (PDB ID: 6TSG) and MMP9 (PDB ID: 8K5Y) proteins.

**Table 5:** Binding free energy of 12 systems calculated by MM-GBSA method.

Systems	Predicted ΔG (kcal/mol) by MM-GBSA method						
	E_vdw_	E_el_	E_gb_	E_surf_	ΔG_gas_	ΔG_solv_	ΔG_total_
TNF-MOL000098	-37.53 ±0.70	-23.31 ±1.42	36.29 ±0.70	-5.36 ±0.05	-60.84 ±1.18	30.93 ±0.67	-29.91 ±0.69
TNF-MOL000354	-37.36 ±0.75	-6.69 ±0.52	25.32 ±0.48	-5.66 ±0.07	-44.05 ±0.95	19.66 ±0.45	-24.39 ±0.80
TNF-MOL000422	-32.70 ±0.61	-17.79 ±1.07	28.59 ±0.53	-4.42 ±0.02	-50.50 ±0.92	24.17 ±0.53	-26.33 ±0.65
IL-6-MOL000098	-27.56 ±1.35	-44.33 ±2.97	51.03 ±1.57	-5.05 ±0.02	-71.89 ±2.34	45.98 ±1.59	-25.91 ±1.09
IL-6-MOL000354	-34.84 ±0.79	-29.41 ±1.75	43.48 ±1.48	-5.63 ±0.04	-64.25 ±1.56	37.85 ±1.47	-26.40 ±0.79
IL-6-MOL000422	-29.24 ±0.57	-19.25 ±0.59	31.21 ±0.65	-4.24 ±0.03	-48.49 ±0.75	26.96 ±0.67	-21.53 ±0.56
PPARG-MOL000098	-36.88 ±0.61	-29.41 ±1.71	38.19 ±1.14	-5.30 ±0.03	-66.29 ±1.23	32.89 ±1.16	-33.40 ±0.38
PPARG-MOL000354	-36.04 ±0.66	-25.56 ±1.93	37.67 ±1.58	-5.30 ±0.04	-61.60 ±1.58	32.37 ±1.59	-29.23 ±0.47
PPARG-MOL000422	-34.97 ±0.42	-8.17 ±0.98	22.25 ±0.36	-5.08 ±0.05	-43.14 ±0.96	17.17 ±0.35	-25.97 ±0.84
MMP9-MOL000098	-36.60 ±0.96	-43.80 ±1.18	58.30 ±0.73	-5.15 ±0.06	-80.41 ±1.45	53.15 ±0.73	-27.26 ±0.88
MMP9-MOL000354	-37.47 ±0.76	-22.24 ±1.29	35.78 ±1.18	-4.91 ±0.06	-59.72 ±1.05	30.87 ±1.19	-28.85 ±0.83
MMP9-MOL000422	-33.70 ±0.58	-25.22 ±1.48	41.08 ±1.29	-4.59 ±0.04	-58.91 ±1.28	36.50 ±1.27	-22.41 ±0.77

**Table 6:** Binding free energy of 12 systems calculated by MM-PBSA method.

Systems	Predicted ΔG (kcal/mol) by MM-PBSA method						
	E_vdw_	E_el_	E_pb_	E_npol_	ΔG_gas_	ΔG_solv_	ΔG_total_
TNF-MOL000098	-37.53 ±0.70	-23.31 ±1.42	54.38 ±0.62	-3.26 ±0.02	-60.84 ±1.18	51.11 ±0.62	-9.72 ±1.28
TNF-MOL000354	-37.36 ±0.75	-6.69 ±0.52	34.27 ±0.79	-3.54 ±0.04	-44.05 ±0.95	30.72 ±0.78	-13.33 ±0.97
TNF-MOL000422	-32.70 ±0.61	-17.79 ±1.07	38.83 ±0.73	-3.34 ±0.01	-50.50 ±0.92	35.49 ±0.73	-15.01 ±0.82
IL-6-MOL000098	-27.56 ±1.35	-44.33 ±2.97	63.63 ±1.90	-3.12 ±0.02	-71.89 ±2.34	60.51 ±1.88	-11.38 ±0.83
IL-6-MOL000354	-34.84 ±0.79	-29.41 ±1.75	55.96 ±1.53	-3.41 ±0.03	-64.25 ±1.56	52.54 ±1.53	-11.71 ±0.76
IL-6-MOL000422	-29.24 ±0.57	-19.25 ±0.59	42.03 ±0.99	-3.03 ±0.04	-48.49 ±0.75	39.00 ±1.00	-9.49 ±0.91
PPARG-MOL000098	-36.88 ±0.61	-29.41 ±1.71	49.61 ±0.90	-3.20 ±0.03	-66.29 ±1.23	46.41 ±0.90	-19.88 ±0.79
PPARG-MOL000354	-36.04 ±0.66	-25.56 ±1.93	52.48 ±2.48	-3.66 ±0.03	-61.60 ±1.58	48.82 ±2.48	-12.78 ±1.60
PPARG-MOL000422	-34.97 ±0.42	-8.17 ±0.98	37.38 ±1.12	-3.27 ±0.03	-43.14 ±0.96	34.10 ±1.11	-9.03 ±1.16
MMP9-MOL000098	-36.60 ±0.96	-43.80 ±1.18	73.24 ±1.16	-3.21 ±0.03	-80.41 ±1.45	70.03 ±1.15	-10.37 ±1.68
MMP9-MOL000354	-37.47 ±0.76	-22.24 ±1.29	43.51 ±1.27	-3.32 ±0.03	-59.72 ±1.05	40.19 ±1.26	-19.52 ±0.98
MMP9-MOL000422	-33.70 ±0.58	-25.22 ±1.48	47.65 ±1.64	-3.15 ±0.03	-58.91 ±1.28	44.50 ±1.65	-14.41 ±1.49

### Anti-liver fibrosis activity in LX2 cells

**Fig 11:**
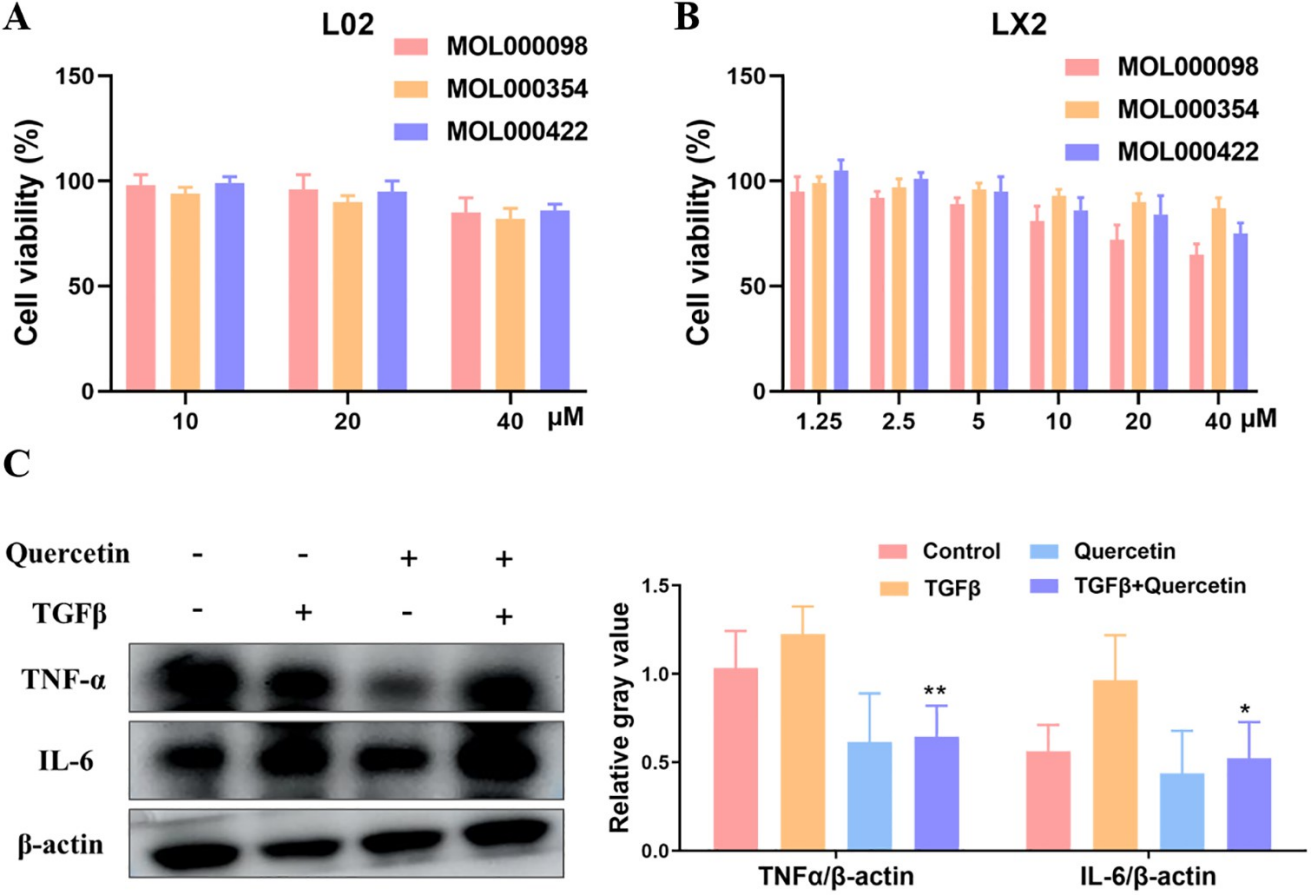
Active components of RB-HMM drug pairs effect on L02 and LX2 cells. (A) (B) Viability of L02 and LX2 cells exposed to 20 ng/mL TGFβ and indicated concentrations of MOL000098 (quercetin), MOL000354 (isorhamnetin) and MOL000422 (kaempferol) measured by MTT assay. (C) The protein levels of TNF and IL-6 were treated with MOL000098 (quercetin) in LX2 cells through western blotting. Image J software was used to analyze the levels of relevant proteins with β-actin as the reference. Data was represented by the mean ±SD of the three independent experiments. **P < 0.01, *P < 0.05, compared with control.

Cell viability assay of L02 and LX2 cells was determined by MTT assay after exposing cells to MOL000098 (quercetin), MOL000354 (isorhamnetin) and MOL000422 (kaempferol) at different concentrations for 72 h. As can be seen from Fig 11A, none of the three compounds showed toxicity to human hepatic cell line L02 compared to control (only cell sap added) for 72 h, suggesting that these compounds might possess safety indexes. Fig 11B illustrates that three compounds could inhibit the viability of human hepatic stellate cell line LX2 to varying degrees and MOL000098 (quercetin) showed more potent toxicity.

Previous studies have shown that TNF and IL-6 were regarded as the top two hub targets for liver fibrosis progression. To investigate whether the active components in RB-HMM drug pairs participate in regulating TNF and IL-6 targets, MOL000098 (quercetin) was selected to treat with the LX2 cell and western blotting assay was performed. LX2 cells were treated with 20 μM MOL000098 (quercetin) for 2 h and then incubated with 20 ng/ml TGFβ for another 72 h. As shown in Fig 11C, the protein levels of TNF and IL-6 were significantly suppressed by MOL000098 (quercetin) in TGFβ stimulated LX2 cells. These results indicated that active components, especially MOL000098 (quercetin), can inhibit cell proliferation and block TNF and IL-6 functions at the cell level.

## Conclusion

Investigating the attributes and contemporary functions of traditional remedies has consistently been a central focus of modern traditional Chinese medicine research. RB and HMM are frequently employed in traditional Chinese medicine for treating liver fibrosis clinically. Nevertheless, the precise mechanisms underlying their combined anti-fibrotic effects remain unclear. This study introduces a novel scientific approach to evaluate the efficacy of multi-component, multi-target drug formulations and identify new therapeutic targets for liver fibrosis. We employed network pharmacology techniques in conjunction with bioinformatics analysis and molecular dynamics simulations to elucidate the molecular mechanisms by which RB-HMM drug combinations are utilized in liver fibrosis treatment. Among the 34 active compounds in RB-HMM, three shared compounds, MOL000098 (quercetin), MOL000354 (isorhamnetin), and MOL000422 (kaempferol), exhibited strong binding activity with four key hub targets (TNF, IL-6, PPARG, and MMP9). Our findings suggest that TNF, IL-6, PPARG, and MMP9 are potential therapeutic targets that influence liver fibrosis progression by regulating inflammatory responses and apoptosis through multiple pathways. This study provides a framework for revealing the modern mechanisms of traditional RB-HMM treatment for liver fibrosis and offers a valuable reference for molecular mechanism research using network pharmacology, bioinformatics analysis, molecular docking, and molecular dynamics simulation techniques in traditional drug studies.

## Supporting information

S1 File Original images for blots and gels requirements. (RAR)

S2 File Minimal data set. (DOCX)

S1 Graphical abstract (TIF)
