# Supplementary material for: Mechanism of Radix Bupleuri and Hedysarum Multijugum Maxim drug pairs on liver fibrosis based on network pharmacology, bioinformatics and molecular dynamics simulation
Source: PLoS One. 2025 Jan 27;20(1):e0318336. doi: 10.1371/journal.pone.0318336 (PMC11771889; doi:10.1371/journal.pone.0318336)
Supplement: S2 File — (DOCX) [file pone.0318336.s002.docx]

**Minimal data set**

(1) The cell viability values (%, including concentrations, means, standard deviations(SD)) of L02 cells determined by MTT assay are listed as follows:

| Concentrations(μM) | MOL000098 | | | MOL000354 | | | MOL000422 | | |
| --- | --- | --- | --- | --- | --- | --- | --- | --- | --- |
| 10 | 96 | 94 | 103 | 96 | 96 | 91 | 99 | 97 | 102 |
| 20 | 89 | 102 | 98 | 92 | 86 | 91 | 92 | 93 | 101 |
| 40 | 85 | 92 | 79 | 81 | 88 | 78 | 84 | 89 | 86 |

The OD values of L02 cells determined by MTT assay are listed as follows: (the control OD values are 1.404, 1.195, 0.932)

| Concentrations(μM) | MOL000098 | | | MOL000354 | | | MOL000422 | | |
| --- | --- | --- | --- | --- | --- | --- | --- | --- | --- |
| 10 | 1.130 | 1.106 | 1.212 | 1.130 | 1.130 | 1.071 | 1.165 | 1.141 | 1.200 |
| 20 | 1.047 | 1.200 | 1.153 | 1.083 | 1.012 | 1.071 | 1.083 | 1.094 | 1.188 |
| 40 | 1.000 | 1.083 | 0.930 | 0.953 | 1.035 | 0.918 | 0.988 | 1.047 | 1.012 |

(2) The cell viability values (%, including concentrations, three independent experiments) of LX2 cells determined by MTT assay are listed as follows:

| Concentrations(μM) | MOL000098 | | | MOL000354 | | | MOL000422 | | |
| --- | --- | --- | --- | --- | --- | --- | --- | --- | --- |
| 1.25 | 95 | 102 | 88 | 99 | 96 | 102 | 105 | 100 | 110 |
| 2.5 | 92 | 95 | 89 | 97 | 93 | 101 | 101 | 104 | 98 |
| 5 | 89 | 92 | 86 | 96 | 93 | 99 | 95 | 102 | 88 |
| 10 | 81 | 88 | 74 | 93 | 90 | 96 | 88 | 91 | 79 |
| 20 | 72 | 79 | 65 | 90 | 86 | 94 | 84 | 93 | 75 |
| 40 | 65 | 60 | 70 | 87 | 82 | 92 | 75 | 70 | 80 |

The OD values of LX2 cells determined by MTT assay are listed as follows: (the control OD values are 0.912, 1.019, 0.948)

| Concentrations(μM) | MOL000098 | | | MOL000354 | | | MOL000422 | | |
| --- | --- | --- | --- | --- | --- | --- | --- | --- | --- |
| 1.25 | 0.912 | 0.979 | 0.845 | 0.950 | 0.921 | 0.979 | 1.008 | 0.960 | 1.056 |
| 2.5 | 0.883 | 0.912 | 0.854 | 0.931 | 0.893 | 0.969 | 0.969 | 0.998 | 0.941 |
| 5 | 0.854 | 0.883 | 0.825 | 0.921 | 0.893 | 0.950 | 0.912 | 0.979 | 0.845 |
| 10 | 0.777 | 0.845 | 0.710 | 0.893 | 0.864 | 0.921 | 0.845 | 0.873 | 0.758 |
| 20 | 0.691 | 0.758 | 0.624 | 0.864 | 0.825 | 0.902 | 0.806 | 0.893 | 0.720 |
| 40 | 0.624 | 0.576 | 0.672 | 0.835 | 0.787 | 0.883 | 0.720 | 0.672 | 0.768 |

(3) The protein levels of TNF and IL-6 in LX2 cells through western blotting are listed as follows:

|  | Control | | | TGFβ | | | Quercetin | | | TGFβ+Quercetin | | |
| --- | --- | --- | --- | --- | --- | --- | --- | --- | --- | --- | --- | --- |
| TNFα/β-actin | 1.17 | 0.84 | 1.25 | 1.18 | 1.15 | 1.41 | 0.75 | 0.38 | 0.81 | 0.84 | 0.64 | 0.55 |
| IL-6/β-actin | 0.69 | 0.44 | 0.61 | 0.69 | 1.23 | 1.04 | 0.72 | 0.36 | 0.37 | 0.57 | 0.39 | 0.71 |
