# Supplementary figures and images for: Mechanism of Radix Bupleuri and Hedysarum Multijugum Maxim drug pairs on liver fibrosis based on network pharmacology, bioinformatics and molecular dynamics simulation
Source: PLoS One. 2025 Jan 27;20(1):e0318336. doi: 10.1371/journal.pone.0318336 (PMC11771889; doi:10.1371/journal.pone.0318336)

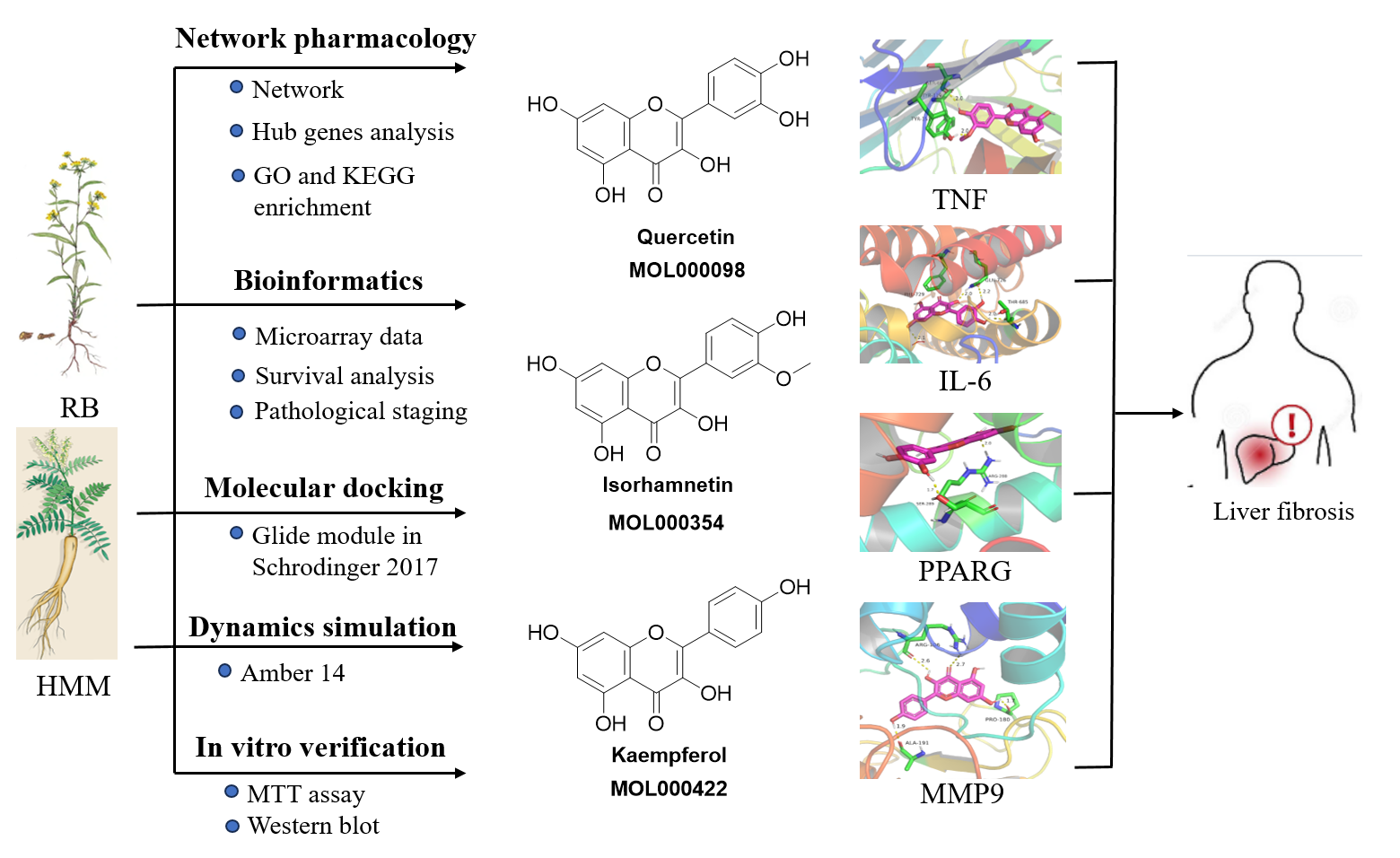

Supplement: S1 Graphical abstract — (TIF) [file pone.0318336.s003.tif]
